# Ferulic Acid Prevents the Hepatotoxicity of AFB1 on Broilers via Regulating Autophagy

**DOI:** 10.3390/vetsci13060549

**Published:** 2026-06-03

**Authors:** Bo Zhang, Lijia Jiang, Yuanyuan Zuo, Hong Zhang, Xinghe Wang, Changde Wu

**Affiliations:** 1 College of Animal Science and Veterinary Medicine, Shenyang Agricultural University, Shenyang 110866, China; zhangbo_0582@163.com (B.Z.); jianglijia0209@163.com (L.J.); zuoyuanyuan1020@163.com (Y.Z.); 2 Petsmate Shennong Hefeng Animal Hospital, Shenyang 110065, China; zhanghonghx@163.com

**Keywords:** ferulic acid, aflatoxin B1, broilers, liver, autophagy

## Abstract

Aflatoxin B1 (AFB1), a common contaminant in broiler feed, impairs hepatic function and poses a significant threat to the poultry industry by disrupting cellular autophagy. Ferulic acid (FA) is a naturally occurring phenolic compound with antioxidant, anti-inflammatory, and autophagy-modulating properties. However, whether FA alleviates AFB1-induced autophagy impairment in broiler liver remains unclear. In this study, an AFB1-induced liver injury model was established. A series of experiments were conducted to evaluate the effects of AFB1 and FA on autophagy-related processes. The results showed that AFB1 exposure was associated with reduced expression of autophagy-related markers, including ULK1, ATG14, ATG5, and LC3-II/LC3-I ratio, suggesting alterations in autophagy-related processes associated with autophagosome formation and maturation. FA supplementation partially reversed these changes and alleviated ultrastructural liver damage induced by AFB1. Transcriptomic and western blot analyses further demonstrated that AFB1 exposure increased p53 expression, dysregulated the BRAF/ERK1/ERK2/mTOR signaling pathway, increased p70S6K expression, and decreased 4E-BP1 expression. FA supplementation alleviated these changes. These results showed that FA may alleviate AFB1-induced alterations in autophagy-related biomarkers in broiler liver, accompanied by changes in the BRAF/ERK1/ERK2/mTOR signaling pathway, suppression of p70S6K expression, and restoration of 4E-BP1 expression, which may contribute to its protective effects against AFB1-induced liver toxicity in broilers.

## Simple Summary

Aflatoxin B1 is a common contaminant in broiler feed. It impairs liver function by disrupting autophagy, thereby threatening broiler health and potentially compromising food safety. Ferulic acid is a naturally occurring phenolic compound widely distributed in medicinal plants. In multiple studies, ferulic acid exhibited its capacity to regulate autophagy. In this study, an aflatoxin B1-induced liver injury model was established to investigate the effects of aflatoxin B1 on different stages of autophagy and the protective effects of ferulic acid. This study suggests that aflatoxin B1 exposure is associated with alterations in several autophagy-related markers involved in different stages of autophagosome formation and maturation. Ferulic acid supplementation partially reversed these alterations and was associated with improved expression of autophagy-related markers. Collectively, these findings suggest that FA may alleviate AFB1-induced liver injury through modulation of autophagy-related processes.

## 1. Introduction

Aflatoxin B1 (AFB1) is a secondary metabolite produced by fungi such as Aspergillus flavus and Aspergillus parasiticus [1]. AFB1 is widely distributed in animal feed and exhibits strong hepatotoxicity. Under certain conditions, when AFB1 intake exceeds the detoxification capacity of the liver, it can impair liver and kidney function, growth performance, reproductive capacity, and immune function in animals. It may even lead to teratogenic and carcinogenic effects [2]. Liver injury in poultry is often accompanied by alterations in hepatic metabolic functions and serum biochemical indices, which can further trigger a range of systemic disorders, including nutritional metabolic imbalance and immunosuppression [3,4]. In addition, differing levels of AFB1 intake and variations in age can lead to broilers developing acute or chronic liver damage following the feeding of AFB1-contaminated feed. This condition also impairs growth performance and reproductive capacity, posing a threat to their health and consequently resulting in substantial economic losses [5].

Autophagy is a highly conserved intracellular degradation process in eukaryotic cells, plays an essential role in maintaining cellular homeostasis [6]. Autophagy plays a vital role in maintaining liver function. Hepatocytes rely on autophagy-derived nutrients and energy to maintain metabolic homeostasis. to sustain normal metabolic functions [7]. However, research has shown that AFB1 inhibits autophagy. AFB1 promotes apoptosis in Leydig cells by inhibiting AMPK/mTOR-mediated autophagy [8]. Our previous study found that AFB1 exposure can affect the expression of autophagy biomarkers in broiler livers, including LC3, Beclin-1, mTOR and cytoplasmic p53 [9]. Therefore, AFB1-induced liver injury may be associated with impaired autophagy.

The autophagy process involves multiple steps, including autophagy initiation, autophagosome formation, autophagosome maturation, and autophagosome-lysosome fusion. Several autophagy-related proteins participate in different stages of this process. ULK1 is essential for autophagy initiation. ATG14 is a pivotal node linking autophagosome formation with autophagosome-lysosome fusion [10]. ATG5 is an essential regulator of autophagosome formation, and the LC3-II/LC3-I ratio is widely used to evaluate the level of autophagy. (Figure S1, Supplementary Materials). Furthermore, the endoplasmic reticulum (ER) is recognized as a major membrane source for autophagosome formation [11]. During autophagy initiation, multiple autophagy-related proteins can be recruited to ER-associated membrane structures, where they assemble the autophagy initiation complex [12,13]. Therefore, assessing autophagy-related markers in both total liver proteins and ER proteins may be essential for evaluating the effects of AFB1 on autophagy-related processes. In addition, autophagy is regulated by multiple signaling pathways, among which mTOR serves as a central negative regulator of autophagy. Suppression of mTOR activity promotes autophagy initiation through activation of the ULK1 complex [14]. Recent studies have shown that p53, as a regulator of stress responses, also plays a key role in the regulation of autophagy. p53 can influence autophagy by modulating the mTOR signaling pathway, and its abnormal expression typically reflects changes in the autophagy state [15]. In the mTOR signaling pathway, changes in its upstream and downstream signals can also influence mTOR activity. Under stress conditions, the activation of ERK1/2 influences mTOR activity, and it has been reported that AFB1 exposure significantly increases the expression of p-ERK1/2 [16]. BRAF functions as an important upstream signaling molecule of ERK1/2, while disruption of mTOR directly influences its downstream signaling molecules, p70S6K and 4EBP1. Since transcriptome analysis in this study identified a differential expression of 4E-BP1, a downstream molecule related to mTOR signaling, the BRAF/ERK/mTOR pathway was selected for further investigation. However, it remains unclear whether this signaling pathway is involved in AFB1-induced inhibition of autophagy in broiler livers.

Ferulic acid (FA) is a naturally occurring phenolic acid widely distributed in plants. It exhibits diverse physiological activities including antioxidant, anti-inflammatory, antibacterial, antithrombotic, immunomodulatory, and anticancer effects [17,18]. Furthermore, studies have demonstrated that FA exerts hepatoprotective effects against a variety of drugs and toxicants [19]. Regarding autophagy, FA counteracts TNF-α/cycloheximide-induced cardiomyocyte apoptosis by regulating autophagy [20]. FA exhibits neuroprotective effects both in vivo and in vitro by activating autophagy to decrease the α-synuclein levels [21]. In addition, FA possesses advantages including low cost, favorable safety, and structural stability. However, it remains unclear whether FA can alleviate the AFB1-induced inhibition of autophagy in broiler livers, especially concerning the effects of AFB1 on the various stages of autophagosome formation. This study aims to investigate the effect of FA on different stages of autophagy in broiler hepatocytes exposed to AFB1.

## 2. Materials and Methods

### 2.1. Animals and Diets

Sixty one-day-old AA broilers were purchased from Shenyang Huamei Livestock and Poultry Co., Ltd., Shenyang, China. and fed an antibiotic-free complete diet provided by the company. All chickens were reared under standard hygienic conditions. Animal welfare and ethical review complied with Shenyang Agricultural University standards, with approval number 202303060. Following 3 days of adaptive feeding, chicks were randomly divided into four groups (n = 15 per group): blank control group (Group C), AFB1 exposure group (Group AFB1), ferulic acid administration group (Group T), and ferulic acid control group (Group FA). Group C received the standard diet; Group AFB1 had feed supplemented with 4 mg/kg AFB1; Group T had feed supplemented with 4 mg/kg AFB1 + 240 mg/kg ferulic acid; and Group FA had feed supplemented with 240 mg/kg ferulic acid. The concentrations of AFB1 and FA used in this study were selected based on our previous studies and preliminary experimental results [22]. The ingredients of the standard broiler diet included 57.12% corn, 27.34% soymeal, 3% cottonseed meal, 2.82% oil, 3% DDGS, 2% corn gluten meal, 0.64% L-lysine, 0.2% DL-methionine, 1.26% calcium hydrogen phosphate, 1.6% stone powder, 0.35% salt, 0.2% choline chloride, and 0.47% additive. Throughout the experiment, all broilers had ad libitum access to feed and water. After 28 days of continuous feeding, blood sampling and euthanasia were performed. Liver tissues were harvested; a portion was fixed in 2.5% glutaraldehyde solution for ultrastructural pathological examination, while fresh liver tissue was used for extraction of hepatic ER proteins, total proteins, and total RNA (AFB1 purchased from Pribolab Pte. Ltd., Singapore; FA purchased from Shanghai Macklin Biochemical Co., Ltd., Shanghai, China).

### 2.2. Ultrastructural Test of Hepatocytes

As reported in our previous study, the preparation methods of transmission electron microscope (TEM) samples are briefly described as follows [9]. Liver tissues were kept in a 2.5% glutaraldehyde, fixed in 1% osmic acid for 2 h, dehydrated in graded ethanol solutions and acetone, and embedded in epoxy resin. The ultra-thin sections (50–70 nm) of liver tissue were stained with a saturated solution of lead citrate in 2% uranyl acetate in 50% ethanol. The ultrastructure of hepatocytes was examined by a TEM (Hitachi High-tech Co. Ltd., Tokyo HT7800, Japan).

### 2.3. RT-qPCR

Liver tissues were ground using a mortar and pestle and disrupted by the addition of TRIzol reagent (Magen Biotechnology Co. Ltd., Guangzhou, China). The total RNA was harvested from the liver tissue and transcribed into cDNA by reverse transcription kits (GenStar Biosolutions Co. Ltd., Beijing, China). The reverse transcription conditions followed the kit instructions. All samples were analyzed in biological and technical triplicates using the 2× RealStar Fast SYBR QPCR Mix kit (purchased from Genstar) in a 20 μL reaction volume consisting of 2 μL cDNA, specific forward and reverse primers (0.5 μL each), and 10 μL 2× RealStar Fast SYBR QPCR Mix. The following thermal cycling parameters were used: 95 °C pre-denaturation for 2 min, 95 °C denaturation for 15 s, 60 °C annealing/extension for 30 s, comprising one pre-denaturation cycle and 40 denaturation and annealing/extension cycles. The mRNA expression levels of *ulk1*, *atg14*, *atg5*, *lc3* were normalized to β-actin. The mRNA expression levels of *p53* were normalized to GAPDH. The relative changes in mRNA gene expression were calculated using the 2^−ΔΔCt^ method. The sequences for all primers are listed in Supplementary Table S1. Primers were designed using Primer Premier 5.0 software and were based on mRNA sequences available at the National Centre for Biotechnology Information (NCBI).

### 2.4. Western Blot Analysis

#### 2.4.1. Hepatic Total Protein Extraction

Liver tissues were homogenized in RIPA lysis buffer (Servicebio, Wuhan, China) and centrifuged at 12,000× *g* for 30 min. The resulting supernatant was collected as the total protein extract. Total protein concentration was determined using a BCA kit (Shanghai Epizyme Biomedical Technology Co., Ltd., Shanghai, China).

#### 2.4.2. Extraction of ER Proteins from Liver Tissue

The extraction of endoplasmic reticulum proteins from liver tissue was performed according to the protocol of the endoplasmic reticulum protein extraction kit (BestBio Biotechnology Co., Ltd., Shanghai, China). Reagent C1 was mixed with C2 to prepare protein extraction solution C, to which a protease inhibitor was added at a ratio of 250:1 immediately prior to use. A total of 500 µL of pre-chilled reagent A was added to the broiler liver tissue and homogenized thoroughly. The homogenate was centrifuged sequentially at 4 °C: 1000× *g* for 5 min, then 11,000× *g* for 10 min. The supernatant was collected after each centrifugation step, whereas the pellet was discarded. The supernatant was centrifuged at 50,000× *g* for 45 min and the pellet was collected. A total of 400 µL. Reagent B was added to the resulting pellet. After thorough mixing, it was centrifuged again at 50,000× *g* for 45 min. A total of 150 µL Protein Extraction Buffer C was added to the final pellet and mixed thoroughly. The centrifuge tube was placed on a shaker at 4 °C for 30 min, intermittently pipetting to ensure homogeneity. The ER protein samples were obtained.

#### 2.4.3. Western Blot Analysis of Autophagy-Related Markers (ULK1, ATG14, ATG5, LC3-I/LC3-II) and Key Targets Associated with Hepatocyte Autophagy Inhibition

The Western Blot method has been reported in our former research [9]. In summary, the primary antibodies: ULK1 (ImmunoWay, Biotechnology Company, Plano, TX, USA, YT4819), 4E-BP1 (Cohesion, Biosciences, London, UK, CPA4399), ATG5 (A19677), p-ERK-1/2 (AP0472) were manufactured by ABclonal, Biotechnology Co., Ltd., Wuhan, China; ATG14 (28021-1-1AP), LC3-II/LC3-I (14600-1-AP), BRAF (20899-1-AP), ERK-1/2 (11257-1-AP), mTOR (66888-1-Ig), p70S6K (14485-1-AP), β-actin (66009-1-Ig), and GAPDH (60004-1-Ig) were manufactured by Proteintech Group, Inc., Rosemont, IL, USA. The horseradish peroxidase-conjugated antibodies (goat anti-rabbit and goat anti-mouse) were manufactured by Elabscience Biotechnology Co., Ltd., Wuhan, China. Antibodies were blocked with rapid blocking solution for 15–25 min. Protein bands were visualized and documented using enhanced chemiluminescence (ECL) reagents. Band intensities were measured with ImageJ software (version 1.54f, National Institutes of Health, Bethesda, MD, USA). Protein expression of ULK1, LC3-I, LC3-II, BRAF, p-ERK1, p-ERK2, ERK1, ERK2, mTOR, p70S6K and 4E-BP1, as well as the LC3-II/LC3-I ratio, were normalized to β-actin, while ATG14 and ATG5 expression were normalized to GAPDH.

### 2.5. Differential Expression Analysis of the Broiler Liver Transcriptome

#### 2.5.1. RNA-Seq

Some differentially expressed genes (DEGs) were identified between the C group the and FA groups. However, no marked changes in autophagy-related molecules were observed. Therefore, the transcriptomic analysis mainly focused on the comparison between the C and AFB1 groups. Transcriptomic sequencing was performed on the C group and the AFB1 group samples; all assays were conducted in triplicate, resulting in a total of six samples. Raw data were obtained from the sequencing machines and filtered. Subsequently, HISAT2 v2.0.5, an upgraded version of TopHat2, was used to align the filtered high-quality sequences (clean data) to the broiler reference genome. Sequencing quality, clean reads, and mapping rates were evaluated before downstream analyses to ensure data reliability. For non-strand-specific libraries, default parameters were applied, whereas for strand-specific libraries, the library type was specified using the appropriate options (--rna-strandness RF for first-strand and FR for second-strand libraries). Gene expression levels were quantified based on the alignment results. Subsequently, further analyses including differential expression analysis, enrichment analysis, and clustering analysis were performed. The successfully mapped reads were assembled to reconstruct the transcript sequences.

#### 2.5.2. Differential Expression Analysis

Differential expression analysis between the control and AFB1 groups was performed using DESeq2 (version 1.20.0). The criteria for screening DEGs were set as *p*-value < 0.05 and |log2 fold change| ≥ 0.58 (fold change ≥ 1.5). Gene Ontology (GO) and KEGG Kyoto Encyclopedia of Genes and Genomes (KEGG) enrichment analysis was performed based on the GO (https://geneontology.org/) and KEGG (https://www.kegg.jp/) databases, respectively. GO enrichment analysis was performed using the topGO package based on the hypergeometric distribution method to identify significantly enriched GO terms among differentially expressed genes. KEGG pathway enrichment analysis was conducted using the clusterProfiler package (version 3.4.4) in R (version 3.5.1). A *p*-value < 0.05 indicated significant enrichment of GO functions. Given the exploratory nature and limited sample size of the transcriptomic analysis, uncorrected *p*-values were used for the preliminary screening of DEGs to reduce the possibility of overlooking potentially relevant genes associated with AFB1 exposure. Meanwhile, adjusted *p*-values (FDR) were also calculated as references for statistical evaluation.

### 2.6. Statistical Analysis

All RT-qPCR and Western blot analyses were performed using biological replicates obtained from different broilers in each group and repeated at least 3 times. The results of the liver index, the mRNA relative expression of ulk1, atg14, atg5, lc3, p53, the differential expression analysis of the broiler liver transcriptome and the relative expressions of ULK1, ATG14, ATG5, LC3-I, LC3-II, BRAF, ERK1, ERK2, mTOR, p70S6K and 4E-BP1 proteins were expressed as the mean ± standard deviation (SD). Prior to statistical analysis, data normality and homogeneity of variance were evaluated using the Shapiro–Wilk test and Levene’s test, respectively. The experimental data were processed and analyzed using SPSS 21.0 software, employing one-way analysis of variance (ANOVA) followed by Duncan’s multiple range test. Bar charts were generated using GraphPad Prism 8 software. *p* < 0.05 and *p* < 0.01 indicate statistical significance (*p* < 0.01 denotes highly significant differences, *p* < 0.05 denotes significant differences).

## 3. Results

### 3.1. Effect of AFB1 and FA Treatment on Broiler Liver Ocular Lesions and Liver Index

**Figure 1:**
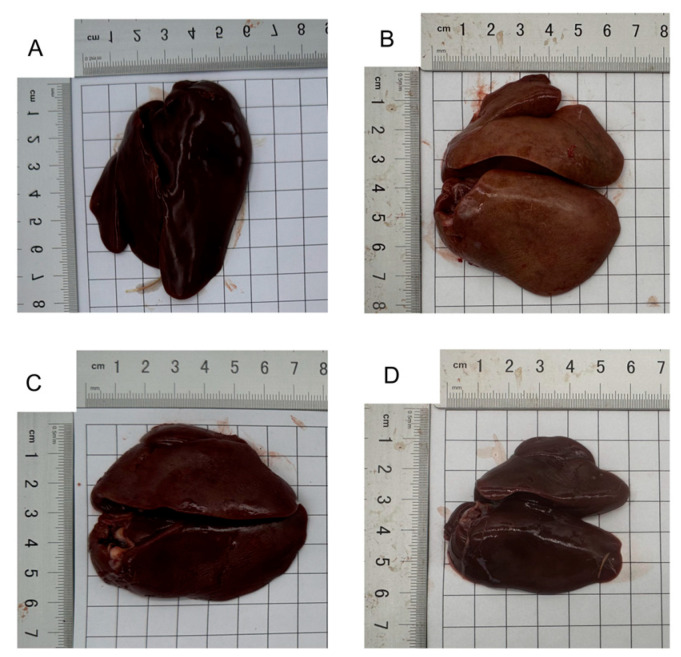
Gross appearance of broiler livers. (**A**) control group; (**B**) AFB1 group; (**C**) FA + AFB1 group (T group); (**D**) FA group.

As shown in Figure 1, the livers of broilers in the C group exhibited a normal reddish-brown color with a soft and smooth surface. Compared with the C group, the broilers in the AFB1 group exhibited a yellowish discoloration of the liver, accompanied by increased fragility. Furthermore, the livers were significantly enlarged. Compared with the AFB1 group, gross pathological lesions in the livers of the T group were markedly reduced.

**Figure 2:**
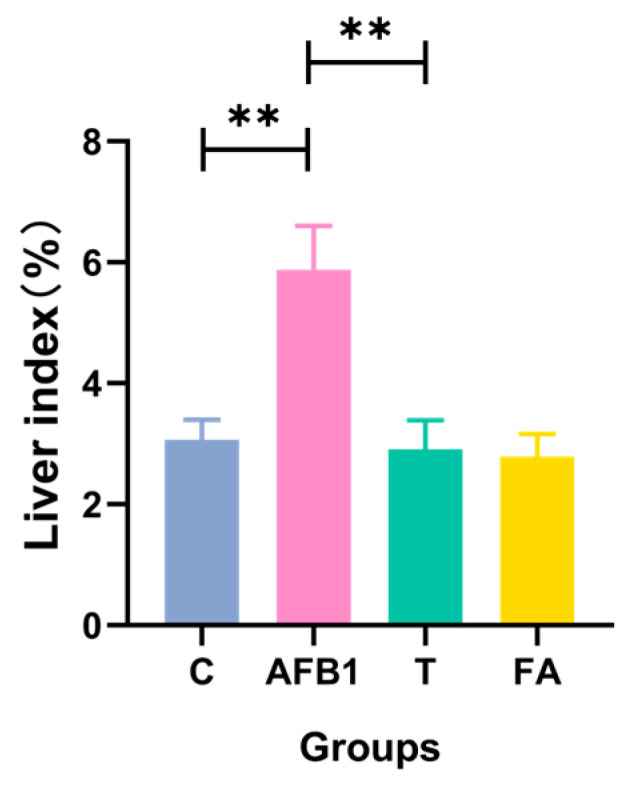
Liver index changes in broilers. The data are presented as mean values ± standard error of the mean (*n* = 15). ** *p* < 0.01.

As shown in Figure 2, the liver index in the AFB1 group was significantly higher than that in the C group (*p* < 0.01). Compared with the AFB1 group, the liver index in the T group was significantly decreased (*p* < 0.01).

### 3.2. Effects of FA on Ultrastructural Changes in Broiler Liver

**Figure 3:**
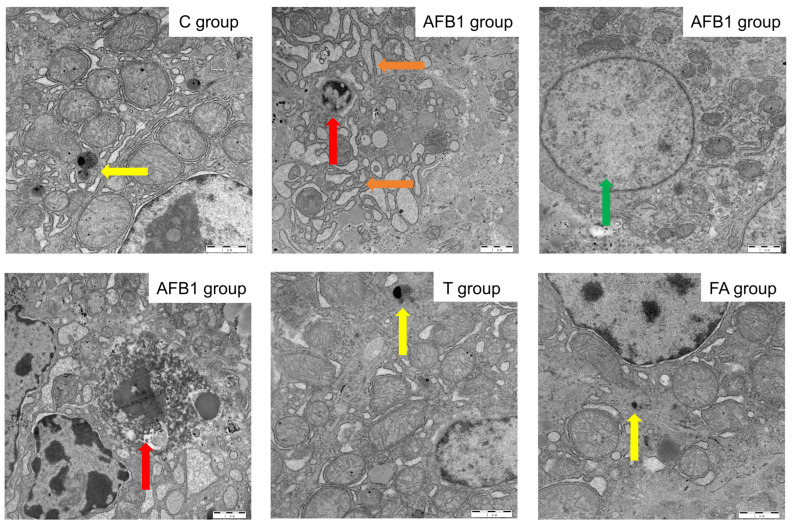
Ultrapathological examination results of broiler hepatocytes. Hepatocytes in the C group, T group, and FA group exhibit normal morphology, shown by yellow arrows. Apoptotic hepatocyte and necrotic hepatocyte in the AFB1 group are shown by red arrows. Abnormal ER structure in AFB1 hepatocytes is shown by orange arrows. Chromatin dissolution and nuclear swelling in the AFB1 group are shown by green arrows.

Figure 3 shows the ultrastructural observations of liver cells from each group of broilers under a TEM. As indicated by the yellow arrows, autophagosomes and autophagolysosomes appeared normal in the liver cells of the C group, T group, and FA group. Conversely, as pointed by the red arrow, hepatocyte apoptosis and necrosis were observed in the AFB1 group. As pointed by the orange arrow, the ER in the hepatocytes was dilated and swollen, and the normal folding structure had disappeared in the AFB1 group. As pointed by the green arrow, chromatin dissolution and nuclear swelling were observed in the AFB1 group. No autophagosome formation was observed in the AFB1 group. These results indicate that FA partially alleviated AFB1-induced hepatocellular ultrastructural damage in broilers.

### 3.3. FA Effectively Alleviates the Down-Regulation of Biomarkers of Autophagosome Formation in Broiler Liver Induced by AFB1

**Figure 4:**
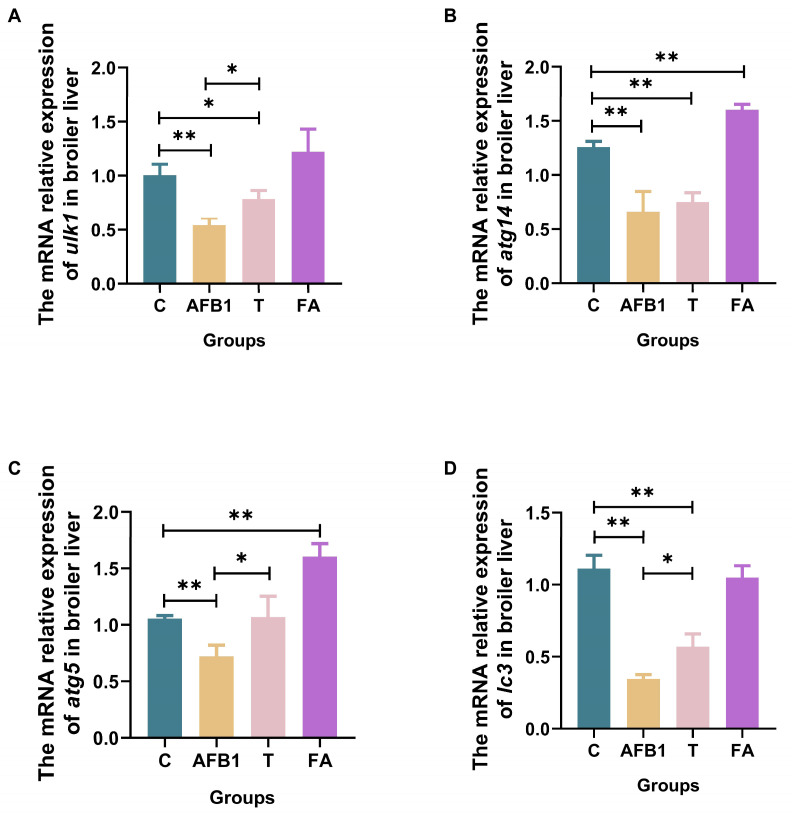
Expression results of biomarker genes at autophagosome formation in hepatocytes of broilers. (**A**) ulk1, (**B**), atg14, (**C**) atg5 and (**D**) lc3. The data are presented as mean values ± standard error of the mean. * *p* < 0.05, ** *p* < 0.01.

AFB1 exposure was associated with changes in autophagy-related biomarkers in liver tissue. As shown in Figure 4A,B, compared with the C group, AFB1 exposure significantly decreased the mRNA relative expression levels of the autophagy precursor conversion biomarkers *ulk1* and *atg14* (*p* < 0.01). As shown in Figure 4C,D, compared with the C group, AFB1 exposure significantly decreased the mRNA relative expression levels of the autophagy formation biomarker gene *atg5* and the autophagy-related biomarker *lc3* (*p* < 0.01). The transcription levels of *ulk1*, *atg5, and lc3* were increased in the T group in comparison to the AFB1 group (*p* < 0.05). In the FA group, the transcription levels of *atg14* and *atg5* were up-regulated compared to the C group (*p* < 0.01).

**Figure 5:**
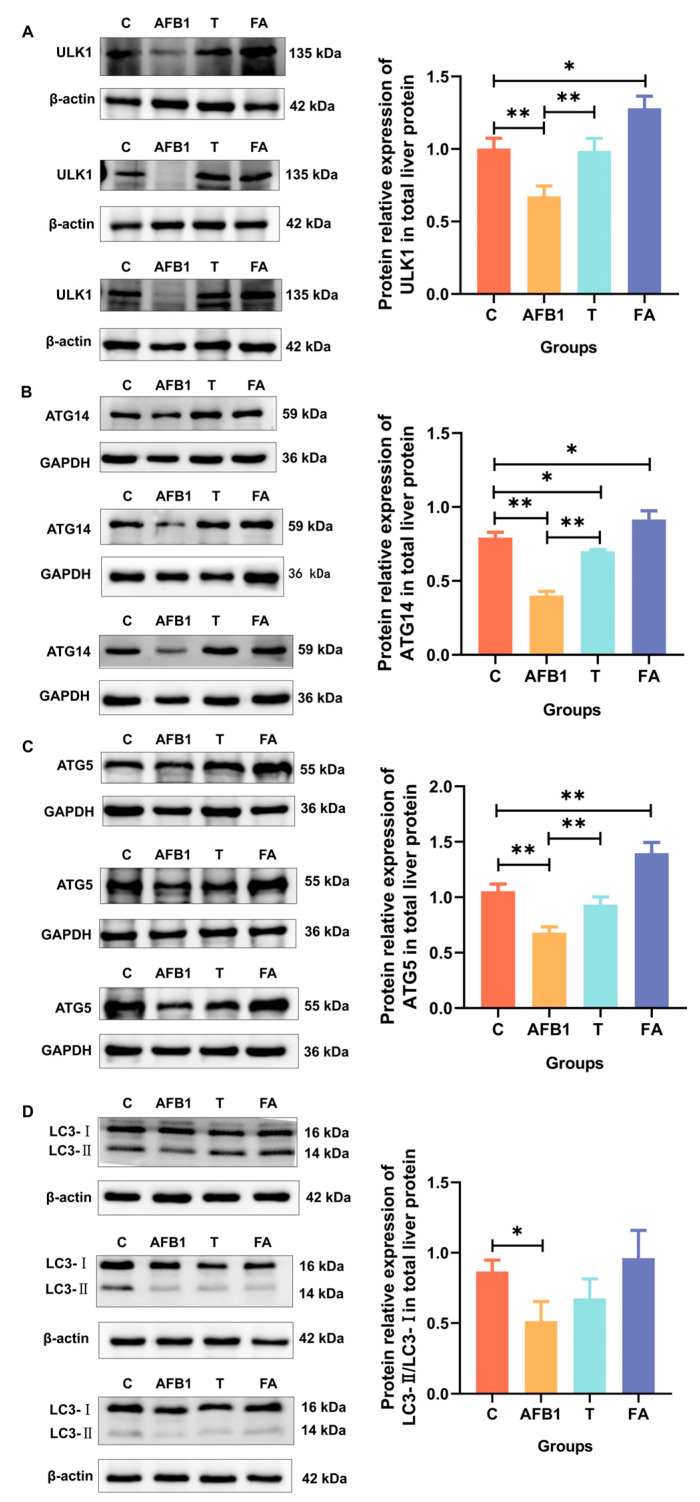
The protein relative expressions of ULK1, ATG14, ATG5 and LC3-II/LC3-I ratio in the total proteins of broiler liver. The relative expressions of ULK1, ATG14, ATG5 and LC3-II/LC3-I ratio were respectively shown in panel (**A**), panel (**B**), panel (**C**), and panel (**D**). Western Blot images were shown on the left. The statistical data were displayed in a column chart on the right. * *p* < 0.05, ** *p* < 0.01.

**Figure 6:**
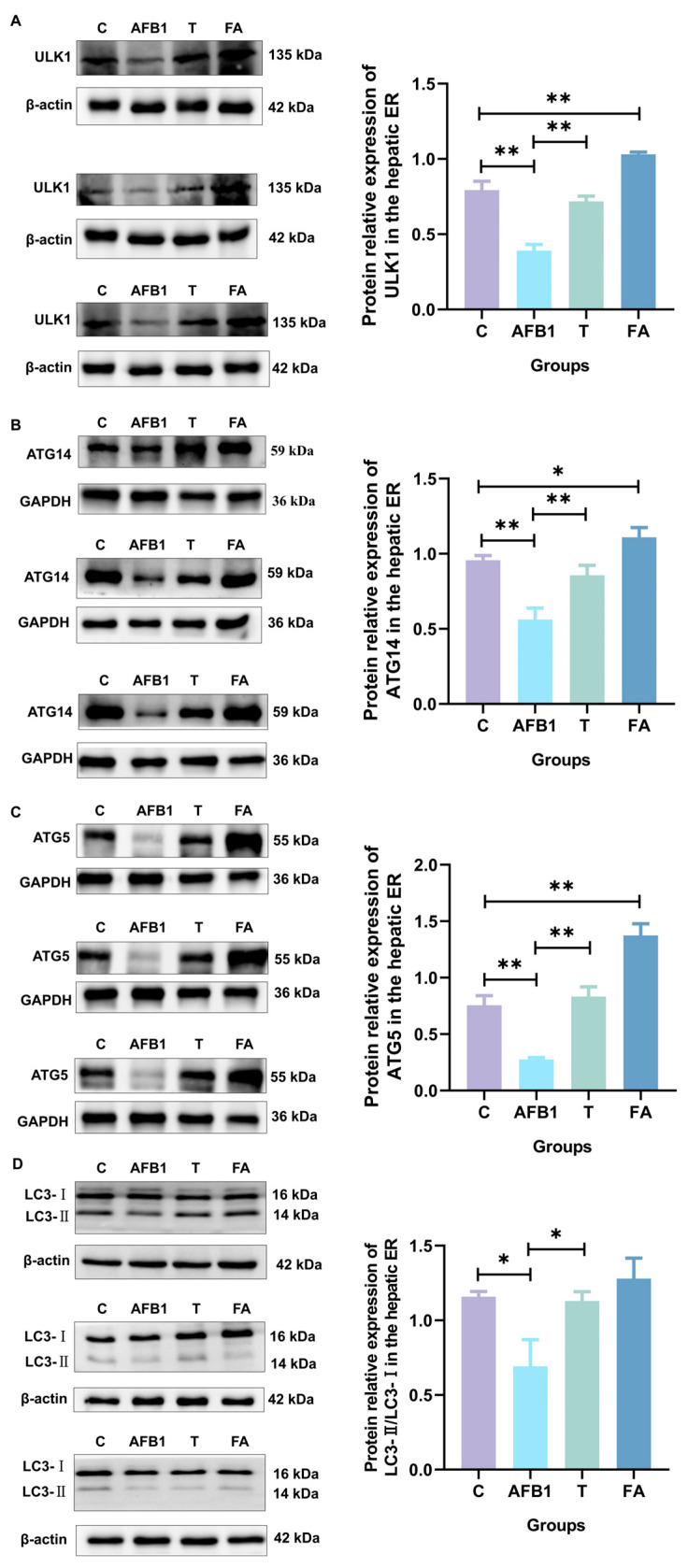
The protein relative expressions of ULK1, ATG14, ATG5 and LC3-II/LC3-I ratio in the ER proteins of broiler liver. The relative expressions of ULK1, ATG14, ATG5 and LC3-II/LC3-I ratio were respectively shown in panel (**A**), panel (**B**), panel (**C**), and panel (**D**). Western Blot images were shown on the left. The statistical data were displayed in a column chart on the right. * *p* < 0.05, ** *p* < 0.01.

As shown in Figure 5 and Figure 6, this pattern was consistent with gene expression levels. Compared with the C group, AFB1-exposed broiler livers showed significantly decreased protein relative expression levels of ULK1, ATG14, ATG5 proteins in both total liver protein and ER protein (*p* < 0.01). In the T group, the protein expression levels of ULK1, ATG14, ATG5 in total liver proteins and ER proteins were significantly higher than in the AFB1 group (*p* < 0.01). As shown in Figure 5, compared with the C group, the LC3-II/LC3-I ratio in total liver protein of the AFB1 group was significantly decreased (*p* < 0.05). Compared with the AFB1 group, the LC3-II/LC3-I ratio in the T group showed no significant difference but exhibited an increasing trend. As shown in Figure 6, compared with the C group, the LC3-II/LC3-I ratio in hepatocellular ER protein of the AFB1 group was significantly decreased (*p* < 0.05). Compared with the AFB1 group, the LC3-II/LC3-I ratio in hepatocellular ER protein of the T group was increased (*p* < 0.05). The expressions of ULK1, ATG14, and ATG5 in total liver protein and ER protein in the FA group were significantly increased in comparison with the control group (*p* < 0.05 or *p* < 0.01).

### 3.4. Identification of Key Targets for AFB1-Induced Autophagy Inhibition in Broiler Liver

#### 3.4.1. Transcriptome Analysis of AFB1-Exposed Broiler Liver

##### Identification of DEGs in AFB1-Exposed Broiler Liver

**Figure 7:**
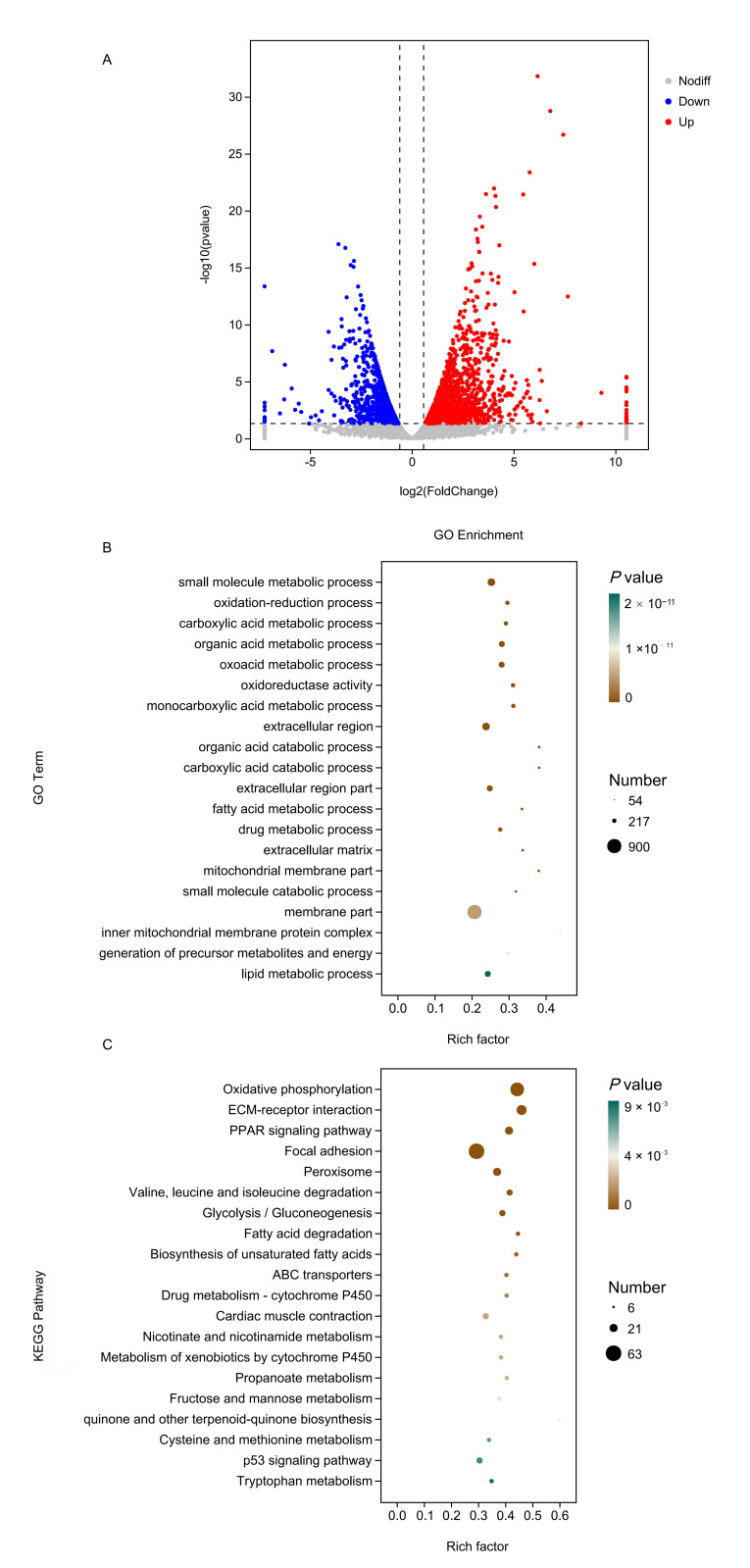
The transcriptome bioinformatics analysis of DEGs in AFB1 exposed broiler liver compared to the control chicken. (**A**) Volcano plot of DEGs; (**B**) GO enrichment analysis; (**C**) KEGG pathway enrichment analysis. DEGs were screened using |log2 fold change| ≥ 0.58 (fold change ≥ 1.5) and *p* < 0.05. *Y*-axis label represents the enriched GO terms or KEGG pathways. *X*-axis label represents the rich factor (rich factor indicates the ratio of the number of enriched DEGs to the total number of genes annotated in the GO terms or KEGG pathways). Bubble size indicates the number of enriched DEGs, while bubble color indicates the enrichment significance level.

As shown in Figure 7A, a total of 3002 DEGs were identified in the comparison between the C group and the AFB1 group. Compared with the C group, 1682 genes were upregulated and 1320 genes were downregulated in the AFB1 group. The statistical results of the differential expression genes analysis are presented in Supplementary Table S2.

##### GO Functional Enrichment Analysis of DEGs in AFB1-Exposed Broiler Liver

Gene Ontology (GO) functions arew divided into three major categories: Biological Process (BP), Cellular Component (CC) and Molecular Function (MF). A GO enrichment analysis for the DEGs and the top 20 GO terms was shown in Figure 7B. GO enrichment analysis showed significant enrichment of several biological processes related to lipid metabolism, mitochondrial function and oxidative stress. Furthermore, studies have indicated that these processes are significantly associated with the regulation of autophagy, which suggests that AFB1 may influence the autophagy process [23,24,25].

##### KEGG Pathway Enrichment Analysis of DEGs in AFB1-Exposed Broiler Liver

KEGG pathway enrichment analysis was performed on the DEGs in the AFB1 group by employing Fisher’s exact test; the top 20 KEGG terms were shown in Figure 7C. The KEGG pathway enrichment results indicate that DEGs are significantly enriched in multiple signaling pathways. Among these, the p53 signaling pathway influences autophagy by regulating the mTOR signaling pathway [26]. The KEGG pathway enrichment analysis suggests that AFB1 exposure may influence the autophagy process.

##### Screening for DEGs Related to Autophagy in AFB1-Exposed Broiler Livers

Through transcriptomic analysis, 4E-BP1 was identified as a DEG associated with the mTOR signaling pathway. Through bioinformatics analysis, and with reference to the KEGG pathway database, the key molecules ERK1 and ERK2 in the 4E-BP1/mTOR signaling pathway were identified, along with the upstream and downstream molecules BRAF and p70S6K. The relevant transcriptomic data are detailed in Supplementary Table S3.

#### 3.4.2. Effects of FA on the Expression Level of Autophagy-Related Genes in Broiler Liver Exposed to AFB1

**Figure 8:**
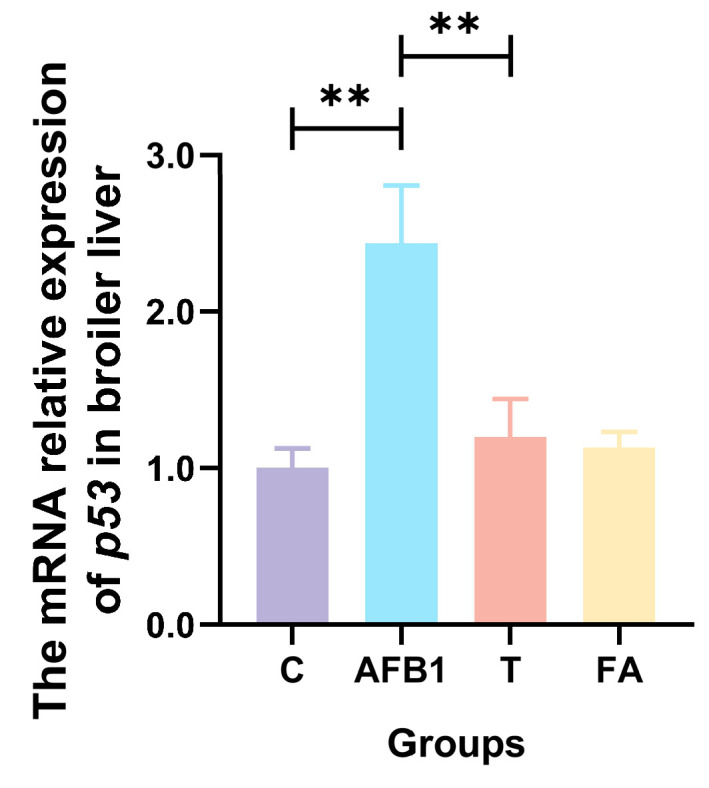
The mRNA relative expression of *p53* in broiler liver. The data are presented as mean values ± standard error of the mean. ** *p* < 0.01.

The results of the KEGG pathway enrichment analysis revealed changes in the p53 signaling pathway, suggesting that it may be involved in the AFB1-induced regulation of hepatic autophagy; therefore, p53 was further examined. As shown in Figure 8, compared with the C group, AFB1 exposure significantly increased the relative mRNA expression of p53 (*p* < 0.01). However, FA supplementation ameliorated the AFB1-induced upregulation of mRNA expression for p53 genes, as evidenced by a significant reduction in relation to the AFB1 group (*p* < 0.01).

#### 3.4.3. Effects of FA on the Expression Levels of Key Proteins Involved in Autophagy in AFB1-Exposed Broiler Liver

**Figure 9:**
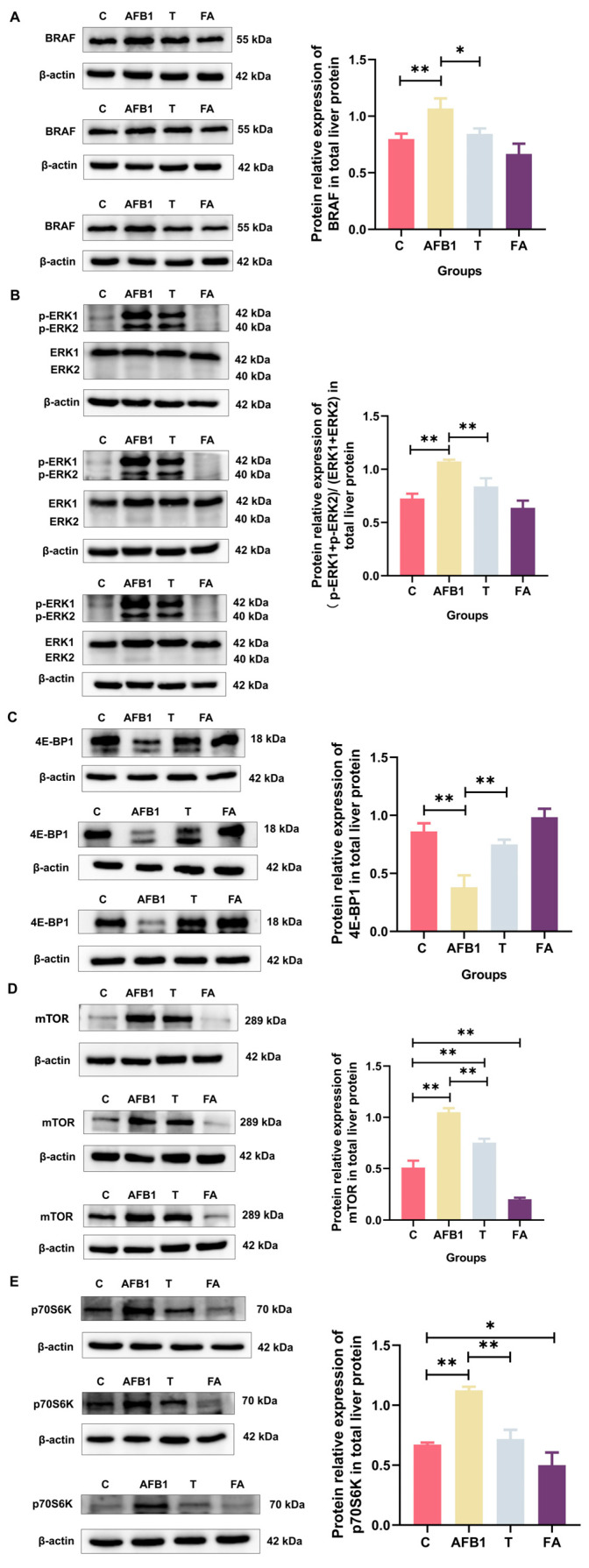
Relative expression of autophagy-related proteins in broiler liver. The relative expressions of BRAF, (p-ERK1 + p-ERK2)/(ERK1 + ERK2), mTOR, p70S6K, and 4E-BP1 were respectively shown in panel (**A**), panel (**B**), panel (**C**), panel (**D**), and panel (**E**). Western Blot images were shown on the left. The statistical data were displayed in a column chart on the right. Asterisk represent statistical difference compared to control group. * *p* < 0.05, ** *p* < 0.01.

As shown in Figure 9, compared with the control group, AFB1 exposure significantly increased the relative expression levels of BRAF, mTOR, and p70S6K proteins, as well as the ratio of (p-ERK1 + p-ERK2)/(ERK1 + ERK2) in broiler liver (*p* < 0.01). Compared with the AFB1 group, the T group showed decreased levels of BRAF, mTOR, p70S6K, and the (p-ERK1 + p-ERK2)/(ERK1 + ERK2) ratio (*p* < 0.05 or *p* < 0.01). The protein expression of mTOR and p70S6K in the FA group was down-regulated significantly in comparison with the C group (*p* < 0.05 or *p* < 0.01). Compared with the control group, the relative expression of 4E-BP1 protein in broiler liver from the AFB1 group showed a significant decrease (*p* < 0.01). Following FA supplementation, the relative expression of 4E-BP1 in Group T was significantly higher than that in the AFB1 group (*p* < 0.01).

## 4. Discussion

AFB1 is currently recognized as the most potent chemical carcinogen discovered. It causes persistent economic losses to the livestock industry by contaminating animal feed during storage and/or transportation [27,28,29]. The liver is the primary target organ for AFB1 [30]. Our previous study has reported that AFB1 impedes the recovery of hepatocyte damage by inhibiting autophagy activity [9]. Therefore, it is imperative to find effective drugs to prevent this damage.

Autophagy, as a crucial process for maintaining cellular homeostasis, is regulated at its various stages by a multitude of key molecules. In this study, changes in several autophagy-related biomarkers, including ULK1, ATG14, ATG5, and LC3-II/LC3-I ratio, were observed following AFB1 exposure. ULK1 is considered a key initiator of autophagy and participates in the formation of the autophagy initiation complex [31]. Therefore, reduced ULK1 expression following AFB1 exposure may reflect suppression of autophagy initiation. ATG14 is a pivotal node linking autophagosome formation to autophagosome-lysosome fusion, suggesting that altered ATG14 expression may be connected with the disruption of autophagosome formation and maturation processes [10]. ATG5 is involved in the formation of autophagosomes [32]. Therefore, the reduction in ATG5 expression following AFB1 exposure may indicate impairment of autophagosome formation. LC3 serves as a pivotal protein in autophagosome maturation. During autophagy, the cytosolic form LC3-I is conjugated with phosphatidylethanolamine to generate LC3-II, which is subsequently recruited to the autophagosomal membrane. The LC3-II/LC3-I ratio is commonly used as an indicator of autophagic activity. Since LC3-II/LC3-I ratio alone cannot fully distinguish autophagosome formation from impaired degradation, the present findings mainly reflect alterations in autophagy-related markers rather than dynamic autophagic flux. In this study, changes in LC3-II/LC3-I ratio further suggested that AFB1 exposure was related to changes in autophagy-related processes. The alterations in these biomarkers indicate that AFB1 exposure may be associated with changes in several autophagy-related processes in broiler liver.

The ER is not only a pivotal organelle for protein synthesis and processing but also serves as a primary membrane source for autophagosome formation [11]. Autophagy, as an important mechanism of cellular self-degradation, is essential for maintaining ER homeostasis [33]. The TEM results in Figure 2. showed that AFB1 exposure led to abnormalities in ER morphology, accompanied by reduced autophagosome formation. In addition, the trends of ULK1, ATG14, ATG5, and LC3-II/LC3-I ratio in ER proteins were consistent with those in total liver proteins, further confirming that AFB1 affects multiple stages of autophagy. Therefore, the changes in these biomarkers within ER proteins suggest that AFB1 may interfere with ER-associated autophagy, thereby disrupting the initiation and progression of autophagy. FA supplementation alleviated this impairment and restored autophagy levels.

mTOR is a central regulator of autophagy. Researchers have demonstrated that AFB1 exposure can activate the mTOR signaling pathway, and that mTOR activation can directly drive the inhibition of autophagy [34,35]. Meanwhile, elevated mTOR activity could inhibit the initiation of autophagy by regulating ULK1. In addition, mTOR can influence the expression of other autophagy-related proteins [36]. Previous studies have shown that p53 inhibits autophagy by activating the mTOR signaling pathway [15]. In the present study, AFB1 exposure was accompanied by increased p53 expression and activation of the BRAF/ERK1/ERK2/mTOR signaling pathway. Screening of transcriptomic data identified 4E-BP1 as a DEG associated with the mTOR signaling pathway, further suggesting that mTOR signaling pathway may be altered during AFB1 exposure. In addition, since BRAF functions as an upstream signal molecule of ERK1/2, can activate ERK1 and ERK2, thereby promoting the high expression of mTOR [37,38]. In the present study, AFB1 was accompanied by increased p53 expression, which positively regulated mTOR activity and consequently suppressed ULK1-mediated autophagy initiation. Meanwhile, Western blot analysis revealed that AFB1 significantly activated the BRAF/ERK1/ERK2 signaling axis, as evidenced by increased BRAF expression and an elevated (p-ERK1 + p-ERK2)/(ERK1 + ERK2) ratio. These changes resulted in increased mTOR activity, indicating that both p53 and BRAF/ERK pathways may contribute, independently or cooperatively, to mTOR signaling dysregulation under AFB1 exposure. Activated mTOR further increased p70S6K expression while decreasing 4E-BP1 levels, which may reflect phosphorylation-dependent inactivation of 4E-BP1, thereby promoting protein synthesis and inhibiting autophagy. The alterations in p53 expression, BRAF/ERK1/ERK2/mTOR signaling pathway, and autophagy-related markers indicate that it may be related to the inhibition of AFB1-induced autophagy.

FA is a natural phenolic compound with antioxidant and hepatoprotective properties [39]. Meanwhile, previous studies have shown that FA can reverse the inhibition of autophagy caused by pathological factors [40]. In the present study, FA supplementation alleviated hepatic damage induced by AFB1 and partially reversed changes in autophagy-related biomarkers. These findings support that FA supplementation may alleviate AFB1-induced autophagy-related alterations, potentially through the regulation of BRAF/ERK1/ERK2/mTOR signaling pathway. Collectively, AFB1 exposure was accompanied by alterations in p53 expression and activation of BRAF/ERK1/ERK2/mTOR-related signaling pathways. These changes are accompanied by modulation of the autophagy-related markers, suggesting that p53 and BRAF/ERK1/ERK2/mTOR signaling pathways may be involved in AFB1-induced inhibition of autophagy. FA supplementation restored the expression levels of these molecules and restored normal autophagy activity.

Several limitations of the present study should be acknowledged. First, transcriptomic analysis was primarily performed to identify autophagy-related molecules and signaling pathways potentially associated with AFB1-induced liver injury through comparison of the C and AFB1 groups. Because of the relatively small sample size, DGE was initially screened using a nominal significance threshold (*p* < 0.05) as an exploratory approach to avoid overlooking potentially relevant targets. Nevertheless, the key genes and pathways highlighted in this study remained significant after FDR correction and were subsequently validated by RT-qPCR and Western blot analyses. Therefore, the transcriptomic findings served mainly as a discovery tool for candidate molecules and were interpreted in conjunction with multiple independent experimental datasets. In addition, autophagic flux assays, pathway inhibition experiments, and multiple-dose FA treatments were not included in the present study. Although alterations in autophagy-related markers, ultrastructural features, and signaling pathways were observed, autophagic flux was not directly evaluated in this study. These findings suggest an association between AFB1-induced liver injury and autophagy-related alterations, while FA may partially reverse these changes. However, markers such as the LC3-II/LC3-I ratio provide only indirect evidence of autophagy and do not fully capture its dynamic nature. Therefore, the present findings should be interpreted as observational evidence rather than definitive proof of regulation of specific stages of autophagy.

Future studies will incorporate larger-scale transcriptomic analyses with increased sample sizes, direct autophagic flux measurements, multiple-dose FA interventions, and targeted inhibition of the BRAF/ERK1/ERK2/mTOR signaling pathway to further clarify how AFB1-induced autophagy-related alterations contribute to liver injury and how FA mitigates these pathological changes.

## 5. Conclusions

In conclusion, this study demonstrates that FA supplementation alleviated AFB1-induced liver injury in broilers and was accompanied by alterations in autophagy-related markers and the BRAF/ERK1/ERK2/mTOR signaling pathway. These findings suggest a potential association between the hepatoprotective effects of FA and autophagy-related processes. However, because autophagic flux was not directly evaluated and the interpretation of LC3-II/LC3-I ratio alone has inherent limitations, the present results should be considered as observational rather than definitive evidence of autophagy regulation. Further studies incorporating direct autophagic flux assays and pathway-specific interventions are required to clarify the underlying mechanisms.

## Data Availability

The raw data supporting the conclusions of this article will be made available by the authors on request.

## Supplementary Materials

The following supporting information can be downloaded at: https://www.mdpi.com/article/10.3390/vetsci13060549/s1, Figure S1: Cellular molecular signaling pathway diagram of autophagy; Table S1: Primer sequences; Table S2: Statistical results of differentially expressed genes in AFB1 group compared to C group in transcriptome analysis; Table S3: Differential expression of 4E-BP1 following AFB1 exposure.

## Abbreviations

The following abbreviations are used in this manuscript:

AFB1	Aflatoxin B1
FA	Ferulic acid
ER	Endoplasmic reticulum
TEM	Transmission electron microscope
DEG	Differentially Expressed Gene

## References

[1] Yunus A.W., Razzazi-Fazeli E., Bohm J. (2011). Aflatoxin B1 in Affecting Broiler’s Performance, Immunity, and Gastrointestinal Tract: A Review of History and Contemporary Issues. Toxins.

[2] Boutefaha Z., Diab K.A., Gheraibia S., El-Nekeety A.A., Belattar N., Hassan M.E., Abdel-Aziem S.H., Hassan N.S., Abdel-Wahhab M.A. (2023). Screening of the phytochemical constituents of Teucrium polium extract and evaluation of their prophylactic role against the oxidative damage and cytotoxicity of Aflatoxin B1 in rats. Toxicon.

[3] Liang Q.H., Liu Q.Q., Tian S.Z., Yao Q.H., Ye X.Q., Liu W.C. (2026). Dietary fucoidan supplementation ameliorates heat stress-induced liver injury in broilers via modulating peroxidation, lipid metabolism, and ferroptosis. Poult. Sci..

[4] Peng Z., Deng J., Xu Z.J., Niu Q.J., Dessalegn L., Refaie A., Sun L.H., Feng Y.P., Liu M. (2025). Hepatoprotective effects of dandelion against AFB1-induced liver injury are associated with activation of bile acid-FXR signaling in chicks. Toxicon.

[5] Umaya S.R., Vijayalakshmi Y.C., Sejian V. (2021). Exploration of plant products and phytochemicals against aflatoxin toxicity in broiler chicken production: Present status. Toxicon.

[6] You H., Wang L., Meng H., Li J., Fang G. (2025). Autophagy: Shedding Light on the Mechanisms and Multifaceted Roles in Cancers. Biomolecules.

[7] Kim E.Y., Lee J.M. (2025). Liver Metabolism at the Crossroads: The Reciprocal Control of Nutrient-Sensing Nuclear Receptors and Autophagy. Int. J. Mol. Sci..

[8] Chen X., Li C., Chen Y., Ni C., Chen X., Zhang L., Xu X., Chen M., Ma X., Zhan H. (2019). Aflatoxin B1 impairs leydig cells through inhibiting AMPK/mTOR-mediated autophagy flux pathway. Chemosphere.

[9] Wang X.-H., Li W., Wang X.-H., Han M.-Y., Muhammad I., Zhang X.-Y., Sun X.-Q., Cui X.-X. (2019). Water-soluble substances of wheat: A potential preventer of aflatoxin B1-induced liver damage in broilers. Poult. Sci..

[10] Wang W., Yu C., Sun F., Wang R., Xia W., Song Q., Zhang H., Jia Z., Zhang M., Wang H. (2026). The ATG14: Multi-layer autophagy control and an emerging therapeutic target in cancer. Apoptosis.

[11] Fujioka Y., N. Noda N. (2025). Mechanisms of autophagosome formation. Proc. Jpn. Acad. Ser. B.

[12] Cillo M., Buonomo V., Vainshtein A., Grumati P. (2025). Autophagy, ER-phagy and ER Dynamics During Cell Differentiation. J. Mol. Biol..

[13] Gómez-Sánchez R., Chumpen Ramirez S., Vargas Duarte P., Hu Y., Mari M., Olschewski K., Hardenberg R., Fromme J.C., Ungermann C., Reggiori F. (2025). Establishment of the phagophore–ERES membrane contact site initiates phagophore elongation. Nat. Struct. Mol. Biol..

[14] Wang Y., Zhang H. (2019). Regulation of Autophagy by mTOR Signaling Pathway. Adv. Exp. Med. Biol..

[15] Xiong H., He T., Tang J., Chu J., He P. (2026). Kaempferol Ameliorates Functional Constipation in Mice by Regulating Autophagy of Interstitial Cells of Cajal via the p53/AMPK/mTOR Axis. Dig. Dis. Sci..

[16] Ge B., Yan K., Sang R., Wang W., Liu X., Yu M., Liu X., Qiu Q., Zhang X. (2024). Integrated network toxicology, molecular docking, and in vivo experiments to elucidate molecular mechanism of aflatoxin B1 hepatotoxicity. Ecotoxicol. Environ. Saf..

[17] Cavalcanti G.R., Duarte F.I.C., Converti A., de Lima A.A.N. (2021). Ferulic Acid Activity in Topical Formulations: Technological and Scientific Prospecting. Curr. Pharm. Des..

[18] Li D., Rui Y.X., Guo S.D., Luan F., Liu R., Zeng N. (2021). Ferulic acid: A review of its pharmacology, pharmacokinetics and derivatives. Life Sci..

[19] Pandi A., Chakraborty B., Sen N., Kalappan V.M. (2025). The hepatoprotective potential of ferulic acid against a spectrum of pharmaceuticals and toxic compounds. Arch. Toxicol..

[20] Li C., Chen L., Song M., Fang Z., Zhang L., Coffie J.W., Zhang L., Ma L., Wang Q., Yang W. (2020). Ferulic acid protects cardiomyocytes from TNF-alpha/cycloheximide-induced apoptosis by regulating autophagy. Arch. Pharm. Res..

[21] Long T., Wu Q., Wei J., Tang Y., He Y.N., He C.L., Chen X., Yu L., Yu C.L., Law B.Y. (2022). Ferulic Acid Exerts Neuroprotective Effects via Autophagy Induction in C. elegans and Cellular Models of Parkinson’s Disease. Oxid. Med. Cell Longev..

[22] Wang X., Li W., Dai J., Jia M., Na L., Xu W., Wu C., Liu M. (2026). Ferulic Acid Alleviates the Hepatotoxicity of Aflatoxin B1 on Broilers by Conjugating and Down-Regulating Chicken CYP1A5 and CYP2W1. Vet. Sci..

[23] Huang J., Zhang C., Huang C., Deng K., Xiao Y., Gao W., Wu M., Lei M. (2025). Mitochondria Metabolism Regulates Glucose-Lipid Homeostasis in Neurodegenerative Diseases. Research.

[24] Jarocki M., Turek K., Saczko J., Tarek M., Kulbacka J. (2024). Lipids associated with autophagy: Mechanisms and therapeutic targets. Cell Death Discov..

[25] Li S., Yuan H., Li L., Li Q., Lin P., Li K. (2025). Oxidative Stress and Reprogramming of Lipid Metabolism in Cancers. Antioxidants.

[26] Li L., Zou Z., Li Q., Zhang K., Su L., Gu Z. (2021). Extranuclear p53 suppresses autophagy through AMPK/mTOR signaling to promote heat stress-induced vascular endothelial cell damage. Nan Fang Yi Ke Da Xue Xue Bao.

[27] Park S., Lee J.Y., You S., Song G., Lim W. (2020). Neurotoxic effects of aflatoxin B1 on human astrocytes in vitro and on glial cell development in zebrafish in vivo. J. Hazard. Mater..

[28] Syraji Y., Jeyaramraja P.R., Mada T., Gobikanila K. (2025). Comprehensive review of aflatoxin contamination, its occurrence, effects, management, and future perspectives. Discov. Food.

[29] Wang T., Cui R., Yu H.F., Yang D., Zhang S., Nie Y., Teng C.B. (2025). The impact of aflatoxin B1 on animal health: Metabolic processes, detection methods, and preventive measures. Toxicon.

[30] Kabali S., Oner N., Kara A., Sogut M.U., Elgun Z. (2026). Alleviation of Aflatoxin B1-Induced Hepatic Damage by Propolis: Effects on Inflammation, Apoptosis, and Cytochrome P450 Enzyme Expression. Curr. Issues Mol. Biol..

[31] Chen M., Hruley J.H. (2025). The human autophagy-initiating complexes ULK1C and PI3KC3-C1. J. Biol. Chem..

[32] Wei F., Wang Y., Yao J., Mei L., Huang X., Kong H., Chen J., Chen X., Liu L., Wang Z. (2024). ZDHHC7-mediated S-palmitoylation of ATG16L1 facilitates LC3 lipidation and autophagosome formation. Autophagy.

[33] Zhao X.Y., Xu D.E., Wu M.L., Liu J.C., Shi Z.L., Ma Q.H. (2025). Regulation and function of endoplasmic reticulum autophagy in neurodegenerative diseases. Neural Regen. Res..

[34] Lin J.X., Xu C.Y., Wu X.M., Che L., Li T.Y., Mo S.M., Guo D.B., Lin Z.N., Lin Y.C. (2023). Rab7a-mTORC1 signaling-mediated cholesterol trafficking from the lysosome to mitochondria ameliorates hepatic lipotoxicity induced by aflatoxin B1 exposure. Chemosphere.

[35] von Wichert L., Stroh A., Witt M., Wanzel M., Mernberger M., Griewing S., Wundisch T., Pfitzner B.M., Teply-Szymanski J., Litmeyer A.S. (2026). mTOR-driven autophagy suppression defines metabolic vulnerability in CDK4/6 inhibitor-resistant HR(+)/HER2(-) breast cancer. Cell Death Dis..

[36] Senapati P.K., Mahapatra K.K., Singh A., Bhutia S.K. (2025). mTOR inhibitors in targeting autophagy and autophagy-associated signaling for cancer cell death and therapy. Biochim. Biophys. Acta (BBA)—Rev. Cancer.

[37] Dunkerly-Eyring B.L., Pan S., Pinilla-Vera M., McKoy D., Mishra S., Grajeda Martinez M.I., Oeing C.U., Ranek M.J., Kass D.A. (2022). Single serine on TSC2 exerts biased control over mTORC1 activation mediated by ERK1/2 but not Akt. Life Sci. Alliance.

[38] Shan K.S., Rehman T.U., Ivanov S., Domingo G., Raez L.E. (2024). Molecular Targeting of the BRAF Proto-Oncogene/Mitogen-Activated Protein Kinase (MAPK) Pathway across Cancers. Int. J. Mol. Sci..

[39] Bhuia M.S., Chowdhury R., Shill M.C., Chowdhury A.K., Coutinho H.D.M., Antas e Silva D., Raposo A., Islam M.T. (2024). Therapeutic Promises of Ferulic Acid and its Derivatives on Hepatic damage Related with Oxidative Stress and Inflammation: A Review with Mechanisms. Chem. Biodivers..

[40] Li Y., Zhao W., Sair A.T., Li T., Liu R.H. (2024). Ferulic acid restores mitochondrial dynamics and autophagy via AMPK signaling pathway in a palmitate-induced hepatocyte model of metabolic syndrome. Sci. Rep..

