# Supplementary material for: Ferulic Acid Prevents the Hepatotoxicity of AFB1 on Broilers via Regulating Autophagy
Source: Vet Sci. 2026 Jun 3;13(6):549. doi: 10.3390/vetsci13060549 (PMC13308477; doi:10.3390/vetsci13060549)
Supplement: Supplementary file 1 [file vetsci-13-00549-s001.zip › vetsci-4334043-supplementary.pdf]

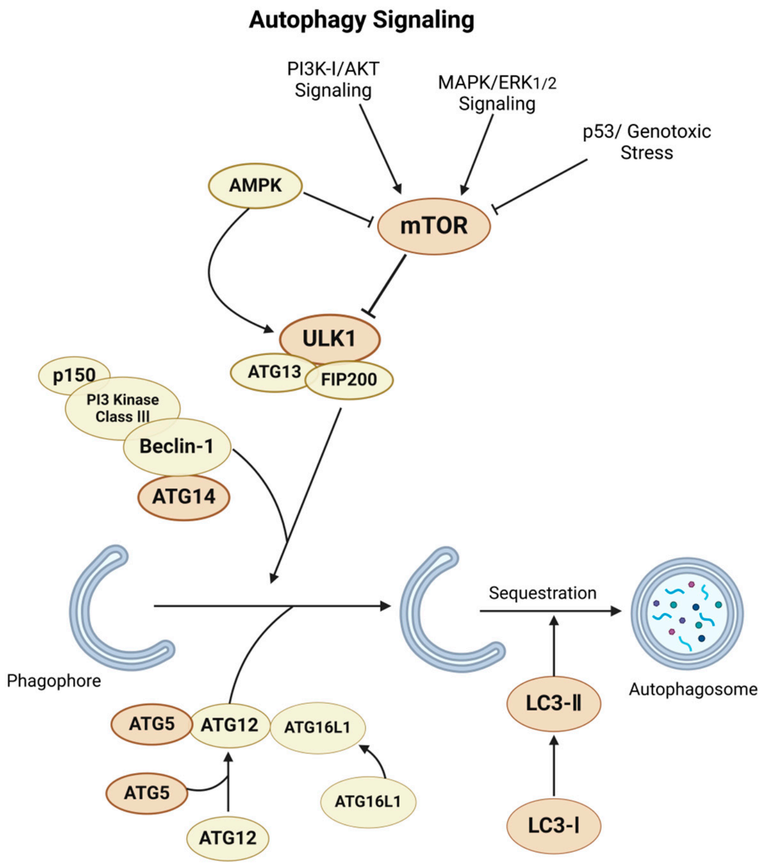


**Supplementary Figure S1.** Cellular molecular signaling pathway diagram of autophagy

**Supplementary Table S1**. Primer sequences

| Sequence Name | Primer sequence (5'-3') | Serial number |
|---|---|---|
| *ulk1* | F: GAGCAAGAGCACACCGACATCC<br>R: TTTCAGGGCAGCAATCTCCATCAC | XM_415091.8 |
| *atg14* | F: GCGACTCCGAGAGGTTTTCA<br>R: TTTGCCTTCCATGGCCTTCA | NC_052536.1 |
| *atg5* | F: GAGGGGTGCTTTCAGTTCCA<br>R: TGAAGCAGGTTGGTATGCGT | NC_052534.1 |
| *lc3* | F: AGTGAAGTGTAGCAGGATGA<br>R: AAGCCTTGTGAACGAGAT | NM_001031461.1 |
| *β-actin* | F: ATGCCATTTTCAGCAGGGCG<br>R: AGATGCAGTGTTGCGAATGTTG | NM_205518.1 |
| *p53* | F: GCCGTGGCCGTCTATAAGAA<br>R: GGTCTCGTCGTCGTGGTAAC | NM_205264.1 |
| *GAPDH* | F: ACTGTCAAGGCTGAGAACGG<br>R: CATTTGATGTTGCTGGGGTC | NM_204305.2 |

**Supplementary Table S2**. Statistical results of differentially expressed genes in AFB1 group compared to C group in transcriptome analysis

| Comparison group | Upregulated of differentially expressed genes | Downregulation of differentially expressed genes | Number of differentially expressed genes |
|---|---|---|---|
| C VS AFB1 | 1682 | 1320 | 3002 |

**Supplementary Table S3**. Differential expression of 4E-BP1 following AFB1 exposure

| Gene | Pathway | BaseMean | BaseMean (Control) | BaseMean (AFB1) | Fold Change (AFB1/Control) | Log2 Fold Change | P-value | FDR |
|---|---|---|---|---|---|---|---|---|
| 4E-BP1 | mTOR signaling pathway | 2610.19 | 3427.54 | 1792.85 | 0.52 | -0.93 | 0.00465 | 0.04861 |
